# Phylodynamic Inference for Structured Epidemiological Models

**DOI:** 10.1371/journal.pcbi.1003570

**Published:** 2014-04-17

**Authors:** David A. Rasmussen, Erik M. Volz, Katia Koelle

**Affiliations:** 1 Biology Department, Duke University, Durham, North Carolina, United States of America; 2 Department of Infectious Disease Epidemiology, Imperial College London, London, United Kingdom; 3 Fogarty International Center, National Institutes of Health, Bethesda, Maryland, United States of America; University of California San Diego, United States of America

## Abstract

Coalescent theory is routinely used to estimate past population dynamics and demographic parameters from genealogies. While early work in coalescent theory only considered simple demographic models, advances in theory have allowed for increasingly complex demographic scenarios to be considered. The success of this approach has lead to coalescent-based inference methods being applied to populations with rapidly changing population dynamics, including pathogens like RNA viruses. However, fitting epidemiological models to genealogies via coalescent models remains a challenging task, because pathogen populations often exhibit complex, nonlinear dynamics and are structured by multiple factors. Moreover, it often becomes necessary to consider stochastic variation in population dynamics when fitting such complex models to real data. Using recently developed structured coalescent models that accommodate complex population dynamics and population structure, we develop a statistical framework for fitting stochastic epidemiological models to genealogies. By combining particle filtering methods with Bayesian Markov chain Monte Carlo methods, we are able to fit a wide class of stochastic, nonlinear epidemiological models with different forms of population structure to genealogies. We demonstrate our framework using two structured epidemiological models: a model with disease progression between multiple stages of infection and a two-population model reflecting spatial structure. We apply the multi-stage model to HIV genealogies and show that the proposed method can be used to estimate the stage-specific transmission rates and prevalence of HIV. Finally, using the two-population model we explore how much information about population structure is contained in genealogies and what sample sizes are necessary to reliably infer parameters like migration rates.

## Author Summary

Mathematical models play an important role in our understanding of what processes drive the complex population dynamics of infectious pathogens. Yet developing statistical methods for fitting models to epidemiological data is difficult. Epidemiological data is often noisy, incomplete, aggregated across different scales and generally provides only a partial picture of the underlying disease dynamics. Using nontraditional sources of data, like molecular sequences of pathogens, can provide additional information about epidemiological dynamics. But current “phylodynamic” inference methods for fitting models to genealogies reconstructed from sequence data have a number of major limitations. We present a statistical framework that builds upon earlier work to address two of these limitations: population structure and stochasticity. By incorporating population structure, our framework can be applied in cases where the host population is divided into different subpopulations, such as by spatial isolation. Our framework also takes into consideration stochastic noise and can therefore capture the inherent variability of epidemiological dynamics. These advances allow for a much wider class of epidemiological models to be fit to genealogies in order to estimate key epidemiological parameters and to reconstruct past disease dynamics.

## Introduction

Genealogies can provide valuable information about the demographic history of a population because the demography of a population can dramatically shape the structure of a genealogy [1], [2]. For example, fluctuations in population size will shift the distribution of branching events, or coalescent times, over a genealogy relative to what would be expected for a population with a constant size [3]. Other aspects of a population's demographic history can also leave behind distinctive genealogical patterns. For example, the structuring of a population into different subpopulations can influence the topology of genealogies, which is often seen as clustering among individuals sampled from the same subpopulation [4]. These observations have led to great interest in statistical methods for inferring demographic trends and parameters from genealogies and given rise to the new field of phylodynamic inference [2], [5]–[8].

Most statistical methods for reconstructing the demographic history of a population from genealogies have been motivated by coalescent theory, which provides a probabilistic framework for relating the demographic history of a population to a genealogy of individuals sampled from that population [9], [10]. Critically, coalescent models provide a way to compute the probability of a given genealogy under a given demographic model. It is therefore possible to estimate parameters of a demographic model, such as population size, from a genealogy using likelihood-based inference methods. Extensions of this basic idea have been used to estimate changes in population size over time, for example by the Bayesian skyline methods available in the BEAST phylogenetic software package [11], [12]. Coalescent theory has also been extended to consider different forms of population structure, giving rise to structured coalescent models [13], [14]. Statistical methods that allow fitting of structured coalescent models to genealogies have the ability to estimate parameters relating to population structure, including migration rates between populations [7], [15].

Recent developments in phylodynamics have focused on developing models and statistical methods for more complex demographic scenarios, which have been largely motivated by the application of coalescent methods to pathogens like RNA viruses with rapidly changing population sizes. For example, coalescent models have been developed for populations where birth (i.e. transmission) rates vary over time [16], [17]. Importantly, the framework of Volz *et al.* [16] also considers the coalescent process in populations where transmission rates change over time in a nonlinear manner, as is often the case for epidemiological models like the well-known Susceptible-Infected-Recovered (SIR) model [18]. Coalescent models have also been developed for common epidemiological scenarios with population structure that alters the rate of coalescence in the population [19], but these models are limited to populations at equilibrium. Finally, Volz [20] presented a framework that brings together both complex population dynamics and population structure. This approach has great appeal as it generalizes coalescent models to allow both birth and migration rates to change over time as a function of the underlying population dynamics, which may be nonlinear and far from equilibrium.

Although recent advances with structured coalescent models have enabled the analysis of more complex epidemiological models, the statistical challenge remains of efficiently fitting stochastic population dynamic models to genealogies. These models can be extremely high-dimensional due to a large number of latent state variables for which we have no direct observations. In Rasmussen *et al.* [21], a particle filtering approach was used to marginalize out these latent variables by forward simulating population dynamic trajectories from the epidemiological model and then averaging over these trajectories to a compute a marginal likelihood. For unstructured models, adapting particle filtering methods to coalescent-based inference is relatively straightforward as the likelihood of a genealogy is simply a function of the simulated population dynamic trajectories. However, for structured models the likelihood also depends on the internal states of lineages in the genealogy, which may change over time as lineages move between populations [20]. The probable state of a lineage can only be calculated retrospectively conditional on the population's demographic history and the state of the lineage at the time of sampling. As we show below, these backward-time dependencies prevent the direct application of forward-time particle filtering methods to structured models.

We therefore present a new statistical approach for fitting stochastic population dynamics models to genealogies using the structured coalescent approach presented in Volz [20] using a modified particle filtering algorithm. This modified algorithm allows for efficient particle filtering under structured coalescent models where the probability that a lineage is in a certain population may depend on both the past dynamics of the population as well as future sampling of lineages. Using this algorithm, we can fit stochastic, nonlinear epidemiological models with essentially any form of population structure to genealogies as long as the model is Markovian. Because population structure arises naturally in many epidemiological models, we define population structure in a very broad sense and consider any model where the population of infected hosts is structured into different nonequivalent states and therefore lineages in different infected hosts do not necessarily have an equal probability of coalescing. This includes models with spatial structure, multiple stages of infection and models of vector-borne and other multi-host pathogens.

The paper has the following structure. First, we present the forward-time epidemiological models that we use as examples throughout the paper. Next, we review the framework first developed in Volz [20] for how coalescent models can be derived for a corresponding forward-time population dynamic model. We then describe how we can fit structured epidemiological models to genealogies given the corresponding structured coalescent model. The statistical method we describe combines MCMC methods with our particle filtering algorithm, and is a variation of the particle MCMC algorithm of Andrieu *et al.* [22]. Using simulated genealogies, we show that this algorithm can accurately reconstruct population dynamics in structured populations and obtain reliable estimates of epidemiological parameters such as transmission rates. We then apply our approach to the HIV epidemic in Detroit, Michigan in order to estimate stage-specific transmission rates and infer how prevalence and incidence have changed over the course of the epidemic. Finally, we explore under what conditions parameters relating to population structure can be inferred from genealogies and how factors such as sample size affect uncertainty in our estimates.

## Methods

### Epidemiological models

In this paper, we use epidemiological models to demonstrate how mechanistic population dynamic models can be fit to genealogies. More specifically, we will consider the type of Susceptible-Infected-Recovered (SIR) models widely used to study the transmission dynamics of infectious diseases [18], [23]. In SIR-type models, the host population is divided into different compartments depending on the host's state (e.g. susceptible or infected). For generality, we let 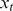 be the vector that holds the number of hosts in each compartment at time 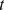, for example 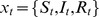 for the standard SIR model. For stochastic models, the state variables in 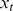 are treated as random variables. We consider an epidemiological model to be structured if there is more than one class of infected host.

#### Applications

We use two simple structured epidemiological models throughout the paper as illustrative examples. The first is a SIR model with three stages of infection, which illustrates how our approach can be applied to models where infected hosts progress through different stages of infection. In the Results section, we apply this model to HIV data so we assume that these three stages correspond to the early, chronic and AIDS stages of HIV infection. The deterministic skeleton of the three-stage SIR model can be written as the following system of ordinary differential equations:
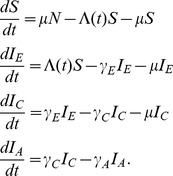
(1)Infections progress from one stage to the next according to the rates 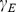 and 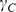. We assume there is no recovery and that individuals with AIDS infection die at rate 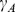 instead of the normal host mortality rate 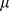, where generally 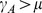.

The force of infection 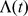 is given by
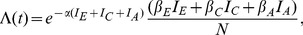
(2)where 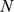 is the host population size (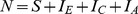). The exponential term in (2) allows incidence to scale nonlinearly with the prevalence of HIV in the population and has been frequently used in HIV models [24]–[26]. This nonlinear scaling may reflect heterogeneity in sexual contact rates or behavioral changes as awareness or diagnosis of the disease grows.

The second model we consider is a simple two-population SIR model where transmission can occur both within and between the two populations due to infectious individuals coming into contact with susceptible individuals in either population. While we do not explicitly define the factor that structures the population, population structure could be due to spatial structure or other factors like age that affect the probability of different hosts contacting one another. The deterministic skeleton of this model can be written as follows:
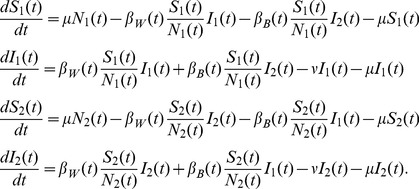
(3)The parameter 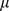 is the host birth/death rate and 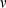 is the rate at which infected hosts recover. 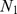 and 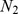 are the host population sizes, respectively. 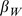 is the within-population transmission rate and 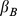 is the between-population transmission rate. We write the transmission rates as 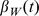 and 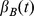 to allow the transmission rate to vary seasonally. Both 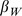 and 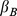 are scaled relative to a base transmission rate 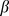 such that 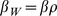 and 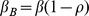, so that the parameter 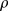 controls the extent of mixing or coupling between the two populations, as in Keeling and Rohani [27]. Under this parameterization, the basic reproductive number 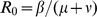 and is therefore invariant to changes in 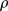 so that we can vary the degree of mixing between populations while not significantly altering the overall epidemiological dynamics.

### Coalescent models

In this section, we consider formulating structured coalescent models for the type of structured epidemiological models just presented. As shown in Volz [20], thinking about population dynamic models as simple birth-death processes can be useful when deriving coalescent models that correspond to a given forward-time model. If we randomly sample individuals from a population and trace their ancestry back in time, then coalescent events in the genealogy will correspond to birth events in the population when both the parent and child lineages are ancestral to sampled individuals. While deaths may affect the overall population size, deaths can be ignored along lineages ancestral to sampled individuals because we know that a lineage could not have died out at an earlier time if it persisted to be sampled at some later time. For a structured population, we also must consider individuals transitioning between different subpopulations through migration events that occur independently of birth events, although for the type of models we will consider here a lineage can also transition between populations by being born into a different population than its parent.

The same birth-death-migration framework can be applied to pathogens if we assume that each infected host corresponds to a single individual in the pathogen population. In this case, births in the pathogen population occur at transmission events between hosts. Deaths in the population will correspond to recovery or mortality of infected hosts. If each infected host is represented by a single pathogen lineage, coalescent events in the genealogy will correspond to transmission events if both the infected host and the infector are sampled or give rise to descendent infections that are sampled. For structured epidemiological models, we also must consider a pathogen lineage transitioning among populations, or compartments in SIR-type models, independent of transmission events. For example, in the three-stage model, pathogen lineages can transition between different stages of infection. Here, we will refer to all transitions between states that occur independently of transmission as migration for generality. This allows many epidemiological models with some form of population structure to be thought of as a birth-death-migration process.

To formalize the birth process, we adopt the notation of Volz [20] and let 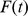 be a matrix that specifies the birth rate of new lineages in the population at time 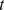, where 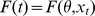, meaning that 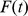 can be a function of the epidemiological parameters 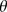 and the population state variables 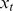. Lineages may be in any one of 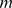 states. The rate at which lineages currently in state 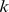 give birth to lineages in state 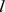 is given by the element 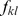. The rate at which migration, or transitions between states independent of birth events, occurs is given by another matrix 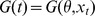. The rate at which lineages currently in state 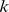 migrate to state 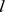 is given by the element 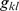. We treat birth and migration as distinct processes because, as we will see, they affect the coalescent process in different ways since coalescent events can only occur at birth events but migration events can affect the probability of a particular lineage coalescing with another lineage. The total number of lineages in each state is given by a vector 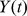, such that 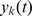 gives the total number of individuals in the population in state 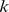 at time 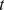. From here in, we drop the time indices and just refer to the matrices 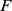 and 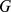 or the vector 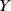, but emphasize that the rates in 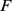 and 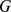 and the population sizes in 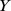 can be time-dependent.

We illustrate the 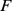 and 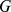 matrix notation by decomposing the three-stage and two-population SIR models presented above into their component birth and migration processes. For the three-stage model, we have
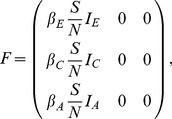
(4)

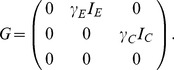
(5)In the 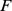 matrix, births occur through transmission of the pathogen from any of the three stages of infection to susceptible individuals. Because all new infections begin in the early stage, only the leftmost column of the 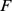 matrix has nonzero elements. The nonzero elements in the 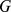 matrix correspond to migration between stages through disease progression from early to chronic and from chronic to AIDS.

For the two-population model, we have
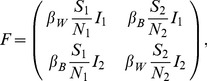
(6)

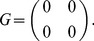
(7)Because transmission events can move the pathogen within and between the two populations in either direction, all entries in the 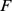 matrix are nonzero. The 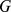 matrix has all zero entries because there is no migration between populations independent of transmission.

Before moving on, we note that for an infectious pathogen our coalescent models make the implicit assumption that coalescent events in the genealogy correspond to transmission events between hosts. In essence then, we are ignoring the within-host coalescent process and assuming that all infected hosts are represented by a single lineage. This implies that lineages immediately coalesce once in the same infected host, which may not be true for certain pathogens where multiple lineages can persist within a host for long periods of time. Nevertheless, in general our assumption that each infected host is represented by a single pathogen lineage will be valid as long as super-infection is rare and there is a strong bottleneck in the pathogen population at transmission events so that it is unlikely that more than one lineage is transmitted between hosts.

### Coalescent likelihoods

To fit a structured coalescent model to a genealogy, we need to compute the likelihood of the coalescent model given the genealogy. To compute this likelihood, we can partition the genealogy into any number of discrete time intervals. We label the time partitioned genealogy 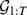, where 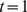 is the time of the first event in the genealogy and 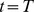 is the final event time going forwards in time (usually the terminal-most sampling event). Time points are chosen to correspond to the times at which events in the genealogy occur such as coalescent and sampling events. We can then further subdivide the genealogy into smaller intervals that correspond to the 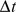 time steps used to simulate from the epidemiological model so that at any time point 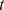 we have the state variables 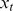 corresponding to that time. With the time partitioned genealogy 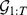, we can compute the likelihood over each interval in the genealogy, 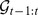, and then take the product over all intervals to compute the total likelihood of the model given 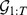.

Computing the likelihood over a time interval 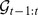 requires us to first compute the probabilities that the lineages present in the genealogy did or did not coalesce within that time interval. The probability of a coalescent event in turn depends on the expected rate of coalescence under the model. This expected rate can be computed for a coalescent model with any arbitrary population structure using the formalism summarized above for the rates of birth in 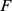. As shown in Volz [20], the rate of coalescence 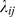 for two lineages 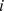 and 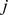 is
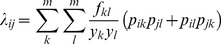
(8)where, for example, 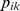 is the probability that lineage 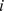 is in state 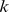. How these lineage state probabilities are computed is explained below. We can make intuitive sense of the coalescent rate in (8) by noting that 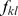 is the total rate at which lineages in state 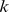 give birth to lineages in state 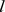 in the population and that 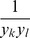 is the probability that lineages 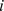 and 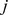 are the two lineages involved in a particular birth event. However, since we do not know the true states of 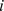 and 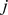 we must sum over all possible combinations of states for these two lineages.

The total rate of coalescence 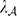 for all lineages 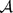 present in the genealogy over an interval of time is then
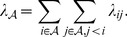
(9)


Given the rates of coalescence, we can then compute the likelihood over a time interval 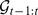 under the coalescent model. If the time interval does not end in a coalescent event, we have
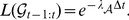
(10)Alternatively, if the interval does end in a coalescent event between two lineages 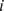 and 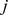, we have
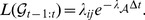
(11)


### Lineage state probabilities

As alluded to above, computing the coalescent rates requires us to compute the probability of each lineage in the genealogy being in each possible state. At the time of sampling, we may know the state of a lineage from information gathered from the infected host from which the sample was obtained. Alternatively, if we do not know the state of the host at the time of sampling exactly, we can assign prior probabilities to the lineage being in each state under a multinomial distribution. Either way, given the initial state or state probabilities at the time of sampling, we need to be able to compute the probability of the lineage being in each state at any point in the past.

Going backwards in time, the lineages transition between states at the rates given in the 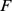 and 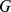 matrices, which in turn depends on the population states 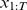 and the parameters 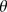. Given these transition rates, we have a continuous time Markov process on a discrete state space along each branch. We can therefore use master equations to track how the lineage state probabilities change going backwards through time. In other words, we can write down differential equations for how the probability mass assigned to each state flows between states as we move into the past. As shown in Volz [20], the general form that these master equations take for any lineage 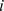 and state 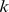 is
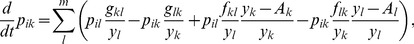
(12)where 
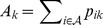
; that is 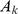 is the expected number of lineages in state 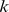 in the genealogy at a given point in time. Further details on how the lineage state probabilities are computed and get updated at coalescent events are given in Text S1. For convenience, we introduce the notation 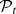 to denote the lineage state probabilities for all lineages in the genealogy at time 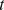 and 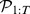 to denote the complete mapping of lineage state probabilities onto the genealogy over the entire time partitioned genealogy 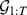.

### Statistical inference

The goal of phylodynamic inference for the type of models presented above will generally be to infer the parameters of interest from the genealogy along with the latent population state variables, such as the number of infected or susceptible hosts over time. In a Bayesian context then, we would like to infer the joint posterior density of the model parameters 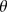 and the latent state variables 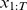. Up to a normalizing constant, this posterior density is given by
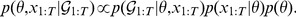
(13)From (13), we see that this joint density can be factored into three parts: the coalescent likelihood 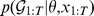 which we outlined how to compute above; the prior density on the population state variables 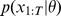 as defined by the epidemiological process model; and the prior density on the parameters 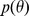. Although we may be able to compute each component individually and thereby the posterior probability of a given set of parameters 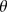 and population states 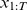, the posterior density is not analytically tractable in general and we must resort to sampling from the posterior using MCMC methods.

However, it may be difficult or impossible to sample from complex, high-dimensional densities such as 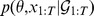 using standard MCMC methods. We could, for example, use a Gibbs sampler to iteratively sample from the conditional posterior densities of 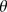 and any component of 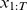, but this strategy can be extremely inefficient owing to strong correlations among the parameters and the state variables, leading to slow MCMC mixing [28]. In Rasmussen *et al.* [21], a particle MCMC approach known as the particle marginal Metropolis-Hastings (PMMH) algorithm was therefore used to sample from the joint posterior density of 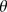 and 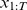. The main motivation behind using the PMMH algorithm is that we can jointly update 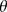 and 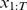 together [22]. Each MCMC iteration, we first propose new parameter values 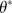 and then run a particle filtering algorithm to get a numerical approximation of the posterior density of the latent state variables 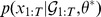, which we refer to as 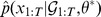. Particle filtering, also known as sequential Monte Carlo, provides a computational means of approximating high dimensional densities by providing samples (i.e the particles) distributed according to the desired density, and are often used in the context of nonlinear and non-Gaussian state space models [29]–[31]. We review how particle filters can be used to fit epidemiological models to genealogies in Text S1.

After running the particle filtering step in the PMMH algorithm, we can then sample a particle from 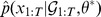 to get a proposal 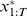 for the latent state variables that is adapted to the parameters in 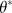. We can also use the particle filter to compute the marginal likelihood of 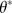 by marginalizing out the state variables. Because we jointly accept 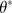 and 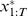 based on the marginal likelihood, we do not have to independently update 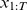, leading to a much more efficient MCMC sampler. Despite marginalizing out the latent state variables, the remarkable feature of the PMMH algorithm is it provides an exact (i.e. unbiased) approximation to the density of interest, 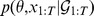. The PMMH algorithm is summarized in pseudo-code below.

#### Algorithm 1

The PMMH sampler targeting 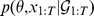


At each MCMC iteration, with current parameter values 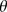:

1. Sample 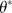 from a proposal density 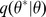.
2. Run particle filter to sample 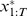 from 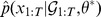 and obtain the marginal likelihood estimate 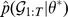.
3. Accept 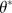 and 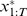 with probability
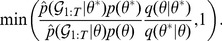
(14)

While the PMMH algorithm described above works for unstructured epidemiological models where all infected hosts are assumed to be in the same population, we encounter an additional problem for structured epidemiological models. In this case, the inference task at hand becomes more difficult because we need to take into account the unknown lineage states. This is done by conditioning on the lineage state probabilities 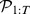 when computing the coalescent likelihood. We can make this dependence on the lineage state probabilities clear by rewriting the likelihood as 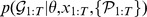. We use the notation 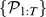 to indicate that while the lineage state probabilities are required to compute the coalescent likelihood, we are not treating the lineage states as random variables but rather as probabilities that are completely determined by the master equations shown in (12) given 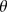 and a population state trajectory 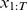.

Recall that the probability of a lineage being in a certain state in the past depends conditionally on the state of the lineage at the time of sampling. This creates a backwards-time dependence structure that cannot easily be accommodated by the forwards in time particle filtering methods used in the PMMH approach. This is because the computational efficiency of the particle filter largely relies on the ability to resample—replacing particles with low weights with particles with high weights. In order to resample, we need to be able to compute the particle weights at any time 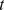, which in turn requires the ability to compute the likelihood 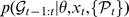 over any time interval. Computing this likelihood therefore entails being able to compute the lineage state probabilities 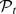, which will depend on the future states of the system 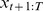 for any structured model. The backward-time dependency of the lineage state probabilities therefore prohibits resampling and thus compromises the efficiency of the particle filter. We therefore use a modified particle filtering scheme that allows us to resample by computing expected lineage state probabilities before running the particle filter and then applying a correction step to counteract any bias introduced by using the expected rather than the true lineage state probabilities while filtering.

In more detail, the algorithm proceeds as follows. We initially simulate a deterministic trajectory from the epidemiological model for the state variables in 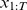, which we refer to as 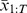. We can then compute the expected lineage state probabilities 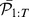 going backwards in time conditional on 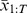. We then run the particle filter forward in time to approximate the density 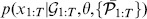. Although 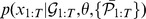 is not ultimately the target density we are interested in, it serves a useful intermediate purpose. Once we have sampled particles representing trajectories from 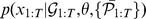, we can re-weight these particles and use an additional round of importance sampling to get particles representing samples from 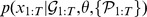, our density of interest.

To do this, for each particle 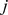 sampled using the particle filter we compute the true lineage state probabilities 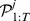 conditional on the actual population state trajectory of the particle 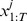. We can therefore compute the corrected weights 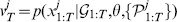 using the true lineage state probabilities. We also store the expected weights 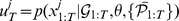 computed using the expected lineage state probabilities. With both the expected weights 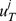 and corrected weights 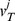 we can assign final importance weights 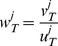 and resample particles again according to the final weights in 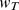. This final round of importance sampling corrects for any bias we may have introduced by resampling particles using the expected lineage state probabilities while filtering and thereby gives us particles approximately distributed according to the target density 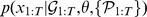.

#### Algorithm 2

The particle filter/importance sampler targeting 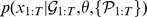

1. Run deterministic simulation to obtain 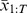 and compute the  expected lineage state probabilities 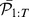 conditional on 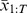.
2. Initialize the particle filter at time 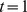 with 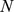 particles.
  - Set 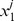 to initial values for all particles.
  - Assign normalized weights, 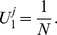

3. Run filter from 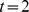 to 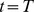.
  - Propagate particles forward by simulating from the process model 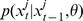.
  - Set 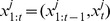 for all particles.
  - Compute unnormalized weights conditional on 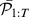,
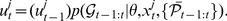
(15)
  - Normalize weights, so that 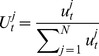.
  - If resampling at 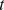, resample according to 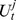.
4. At time 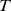, resample particles again according to 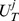 to get particles distributed according to 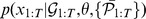.
5. Compute corrected lineage state probabilities 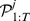 for each particle conditional on 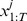.
6. Compute corrected weights 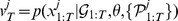.
7. Compute final importance weights 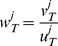 and normalize to get 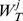.
8. Sample 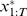 from 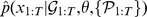 according 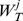.
9. Compute marginal likelihood estimate
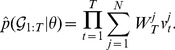
(16)

The particle filter/importance sampler therefore provides us with a proposal for the population state variables 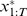 and an estimate of the marginal likelihood. We can therefore plug the particle filter/importance sampler into step 2 of Algorithm 1 to obtain a PMMH algorithm for sampling from the joint posterior density of 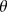 and 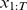 under structured epidemiological models. Moreover, because we marginalize over the population state variables 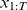 using the particle filter and then marginalize over the lineage states by summing over all possible lineage states when computing the likelihood, we can efficiently sample from the posterior density using the PMMH algorithm without having to design proposal updates for the population states or lineage states.

Before moving on, we make a few notes about the potential limitations and efficiency of the particle filter/importance sampler. As a basic requirement of importance sampling, the support of the importance density 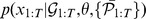 must span the support of the target density 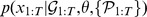), so that wherever 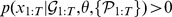 so must 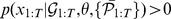. However, in order for the particle filter/importance sampler to be efficient, the density 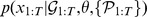 should also be similar to 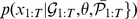. Of course, this might not always be the case. If the stochastic particle trajectories can diverge largely from 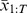 or the lineage state probabilities are highly correlated with 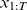 (such that small changes in the population states lead to large jumps in the lineage state probabilities), then these two densities may be quite different, causing the importance sampler to become very inefficient and requiring us to sample many particle trajectories in order to obtain a reasonable particle approximation to 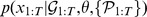. In such cases, it may be unwise to resample particles according to their expected weights 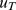 during the particle filtering stage because these weights will not be predictive of the corrected weights 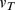, meaning we may be discarding particles with high posterior probability under the desired density 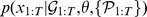. In practice, this can easily be checked by making sure that there is a strong positive correlation between the expected and corrected weights. We found this to be true for all cases considered in this paper and found that resampling according to the expected weights during the filtering stage measurably improved the performance of the algorithm by reducing the variance in the marginal likelihood estimates, which tends to improve MCMC mixing overall.

### Simulations

We simulated mock genealogies under each model to test the performance of the PMMH algorithm before applying the method to real data. Mock genealogies were obtained by first forward simulating from the population dynamic model while tracking all infected hosts in the population and the parent-offspring relationships at transmission events. From the forward simulations, we could then trace the lineages of infected individuals backwards through time to obtain the true genealogy for a fraction of sampled lineages. All population dynamic simulations were performed using the tau-leaping algorithm so that the epidemiological dynamics included demographic noise [32].

The three-stage model was parameterized to reflect the natural history of HIV because we planned to apply our method to real HIV genealogies (see Table 1). We set the disease progression and AIDS death rate to values that give an average time between infection and death of about 10 years, consistent with observed patterns. The incidence scaling parameter 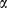 was set to zero so that in the simulations there was a linear scaling between incidence and prevalence. The epidemic simulations were seeded with one early-stage infection at time zero and run for 37 years to reflect the timespan of the HIV epidemic in the U.S. To obtain mock genealogies from the complete infection trees, we sampled 200 individuals in the last six years of the epidemic to reflect the fact that most HIV sequences have been sampled in the recent past. For all parameters, we chose to use uniform priors over a wide range of biologically plausible values so that the choice of prior would have minimal influence on our estimates.

**Table 1:** Fixed parameters in the epidemiological models.

Three-stage model		Two-population model	
Initial population size	*N* = 10,000	Initial population sizes	*N*_1_ = *N*_2_ = 2 million
Birth/death rate	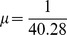 yr^−1^	Birth/death rate	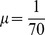 yr^−1^
Progression rate	*γ_E_* = 1 yr^−1^	Recovery rate	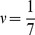 day^−1^
Progression rate	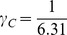 yr^−1^	Seasonal amplitude	*α* = 0.08
AIDS death rate	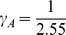 yr^−1^	Seasonal phase	*δ*_1_ = 0.0 yrs
			*δ*_2_ = 0.5 yrs

For the two-population model, we added seasonality to the model by seasonally forcing the base transmission rate 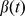 using a sinusoidal forcing function, where
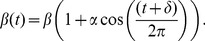
(17)The strength of seasonality 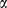 was the same in both populations but we allowed 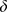 to differ between the two populations to get asynchronous dynamics between populations. The values of all fixed parameters in the model are also shown in Table 1. For the genealogies, 120 infected hosts were randomly sampled over time with sampling effort proportional to disease prevalence in each population. For the two-population model, we fixed the initial conditions for the number of susceptible and infected hosts in each population.

For the simulation experiments, we wished to compare estimates obtained by fitting stochastic models using the PMMH algorithm against estimates obtained by fitting deterministic models. To fit deterministic models, we used a Metropolis-Hastings sampler where, whenever new parameters are proposed, the likelihood of the genealogy under the new parameters is computed by conditioning on a deterministic trajectory of the state variables 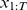 simulated from the model using these new parameters.

### HIV data

We applied our method to a set of HIV-1 partial *pol* sequences collected from men who have sex with men (MSM) in the metropolitan area of Detroit, Michigan. The dataset contained 437 HIV-1 subtype B sequences which were originally collected for drug resistance testing between 2004 and 2011. More information about this dataset can be found in Volz *et al.* [26]. Data were anonymized by staff at the Michigan Department of Community Health before being provided to investigators. Because this research falls under the original mandate for HIV surveillance and was de-identified, it was classified as human subjects research but was exempt from further IRB review.

We reconstructed time-scaled genealogies from the HIV sequences in BEAST using a relaxed molecular clock [33]. All sequences identified as likely recombinants were removed from the alignment prior to the analysis. Tips in the genealogy corresponding to sampled infected individuals were assigned prior probabilities of being in each infection stage based on the time since infection estimated from CD4 cell counts and genetic diversity within the host [34].

From the HIV genealogies, we estimated the transmission rates 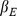, 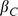 and 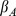 as well as the incidence scaling parameter 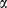. All other parameters were fixed at the values given in Table 1. Rather than estimate initial conditions, the time of the initial introduction of HIV into Detroit was estimated, at which point the epidemic was seeded with one early-stage infection in a completely susceptible population. All priors on the parameters were uniform. For the time of initial introduction the prior was truncated at 1973 as a lower bound and the root time of each tree as an upper bound. To ensure our phylodynamic estimates of HIV incidence were reasonable, we compared our estimates against incidence back-calculated from Michigan Department of Community Health surveillance data using the method of Yan *et al.* [35].

### Implementation

For all results shown in this paper, the PMMH algorithm was run for at least 100,000 iterations or until the MCMC fully converged. For the Metropolis-Hastings step, we chose a multivariate normal proposal density for 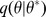, which can take into account the correlations among different parameters by optimizing the covariance parameters that specify the density.

For the particle filter, we found that using a small number of particles (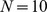) was sufficient. Running the particle filter with a small number of particles tends to increase the error, or variance, in the marginal likelihood estimates. However, this error will not affect inference as long as the marginal likelihood estimates are not systematically biased because the error in the estimates will get averaged out in the encompassing MCMC algorithm. Nevertheless, with too few particles we run the risk of the MCMC getting stuck at erroneously high values of the likelihood. Our choice of 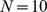 was therefore a compromise between minimizing the error in the marginal likelihood estimates and the time taken to run the particle filter. Resampling within the particle filter was done by multinomial sampling with replacement. Resampling times were chosen to minimize the variance in the marginal likelihood estimates and were usually placed around coalescent events, as most of the variation in particle weights arises at coalescent times.

The PMMH algorithm was implemented in the software package PHYLter and Java source code is freely available at http://code.google.com/p/phylter/. Running the PMMH algorithm for 100,000 iterations using the simulated HIV genealogies took approximately 10 hours (0.36 s per iteration) on a 3.4 GHz Intel i7 processor without any parallelization across cores. The most computationally intensive component of the algorithm is computing the lineage state probabilities, which involves numerically solving the master equations for each lineage in the genealogy and has a time complexity of 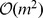, where 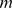 is the number of possible lineage states. On the other hand, run times scale linearly with the number of particles and lineages in the genealogy. Thus, the efficiency of the algorithm is mainly limited by the number of states in the model.

## Results

### Testing the algorithm

**Figure 1:**
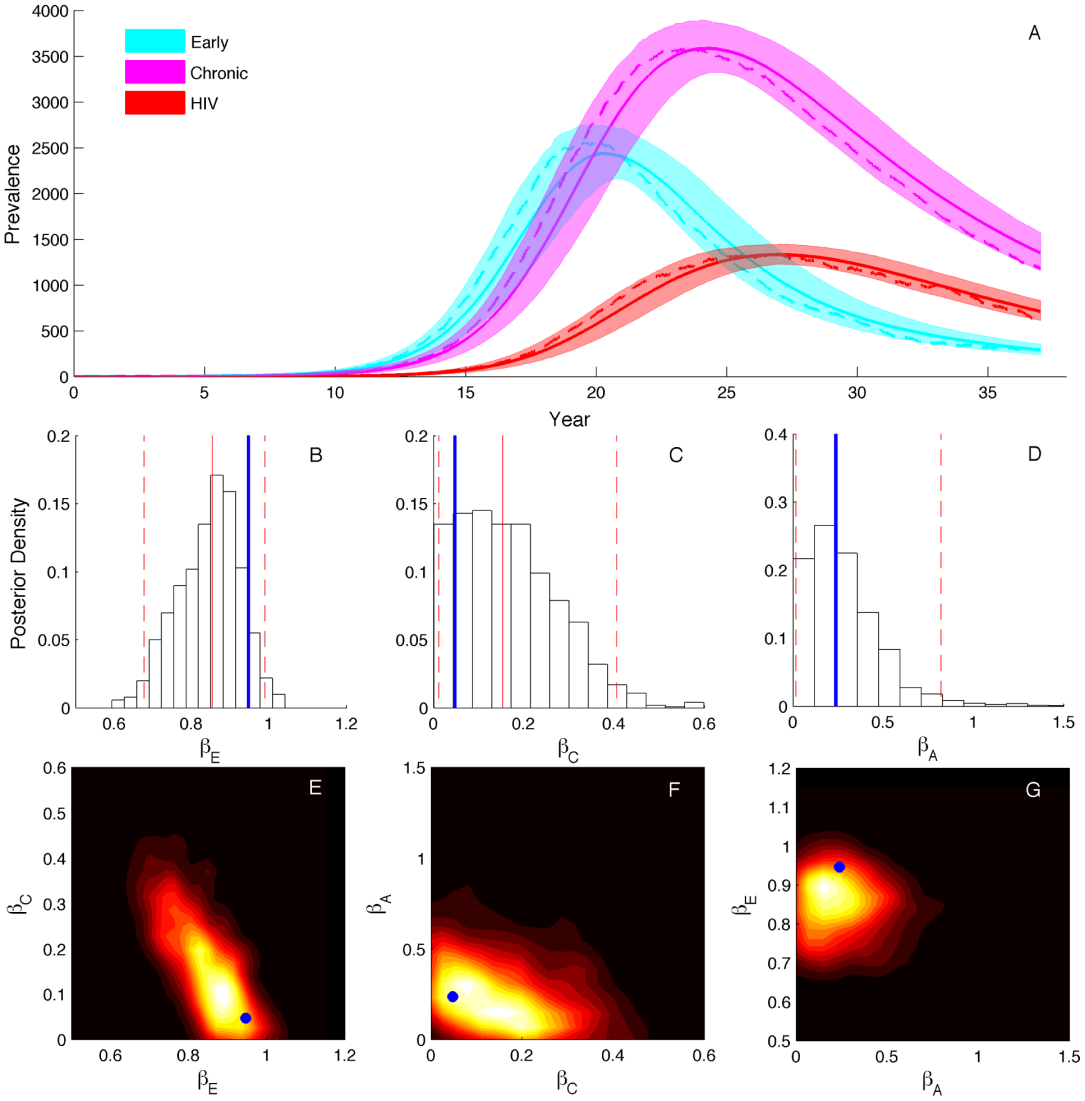
Prevalence and transmission rates estimated from a representative genealogy simulated under the three-stage SIR model. (A) Stage-specific prevalence estimates with the 95% credible intervals shaded and the posterior medians shown as solid lines. Dashed lines show the true prevalence. (B–D) The marginal posterior densities of the stage-specific transmission rates. (E–G) The corresponding pairwise joint densities of the transmission rates, which were constructed from the MCMC samples using nonparametric kernel density estimation.

Before applying the PMMH algorithm to genealogies reconstructed from real data, we ran extensive simulations to ensure that we could accurately recover epidemiological parameters and population dynamics from mock genealogies. We simulated 100 stochastic realizations of an epidemic from the three-stage model, keeping track of the underlying infection tree so that we could obtain the true genealogy for a fraction of sampled lineages. From the simulated epidemic dynamics, we can see that demographic stochasticity generates considerable variation in when the epidemic begins and peaks (Figure S1). Even with this variability, we accurately inferred stage-specific prevalence and transmission rates from the mock genealogies using the PMMH algorithm (Figure 1). The 95% credible intervals generally contained the true prevalence for all three stages of infection (Figure 1A). We were also able to estimate the stage-specific transmission rates associated with each stage of infection (Figure 1B–D), even though there were strong correlations among the different transmission rates as seen in the pairwise joint posterior densities (Figure 1E–G). Overall, out of all 100 simulations, the 95% credible intervals contained all three transmission rates 94 times, while the posterior coverage was greater than 95% for each parameter individually. In contrast, when we fit deterministic models to the same set of genealogies, the credible intervals contained the true parameters only 79% of the time. The PMMH algorithm therefore appears to give reliable estimates of parameters and epidemiological dynamics and outperforms deterministic methods when stochasticity plays a role in the epidemic dynamics.

### HIV in Detroit

Given that we were able to reliably estimate transmission parameters and prevalence in our simulation study, we next applied the method to HIV genealogies reconstructed from sequences collected in Detroit, Michigan. A critical question in HIV epidemiology is to what extent transmission during the early stages of infection contributes to overall HIV incidence. Transmission during early infection may influence the effectiveness of interventions based on antiretroviral treatment in limiting the epidemic [36], [37]. If most new cases of HIV result from recently infected individuals, then prevention strategies that rely on treating diagnosed individuals, who are likely in later stages of infection, will directly prevent few transmissions. Thus, the transmission rate from early HIV infections (EHI) is a key parameter of great interest, although difficult to measure directly from traditional surveillance data. Phylogenetic studies of HIV have used the high degree of clustering and short branch times within these clusters to argue for a high EHI transmission rate [4], [38]. However, clustering alone cannot be taken as definitive evidence for high EHI transmission as similar patterns can arise simply from epidemic transmission dynamics [26]. In this section, we demonstrate that our inference framework can be used to estimate the EHI transmission rate and the number of new HIV infections attributable to EHI from HIV genealogies using models that explicitly consider HIV's transmission dynamics, as well as the stochastic nature of the epidemic dynamics.

Time-scaled genealogies were reconstructed using BEAST from HIV-1 partial *pol* sequences isolated from men who have sex with men (MSM) in the metropolitan area of Detroit. A representative genealogy randomly sampled from the BEAST posterior is shown in Figure S2. We then fit our three-stage SIR model to 10 genealogies sampled from the BEAST posterior to take into account uncertainty in the genealogy. From these genealogies, we estimated the transmission rate for each stage, including the EHI transmission rate, along with the stage-specific dynamics of prevalence and the incidence (i.e number of new cases) attributable to each stage over the course of the epidemic.

**Figure 2:**
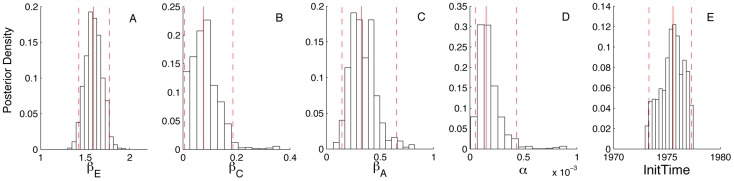
Posterior densities of parameters inferred from one HIV genealogy. Solid red lines mark the median values and dashed lines indicate the 95% credible intervals. The estimated parameters are the early stage transmission rate 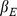, the chronic stage transmission rate 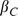, the AIDS stage transmission rate 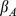, the incidence scaler 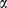 and the initial introduction time of HIV into Detroit.

Parameters estimated from the representative HIV genealogy are shown in Figure 2 and estimates from all 10 genealogies are given in Table 2. We estimated that transmission rates are higher during the early and AIDS stages than during the chronic stage, as expected from previous studies [39]–[41]. The transmission rate from EHI is about 20 times higher than during the chronic stage and about five times higher than during the AIDS stage (Figure 2A–C). We also found evidence for a nonlinear dependence of incidence on prevalence, quantified through the incidence scaling parameter 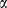. Although estimated values of 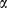 are small, the posterior density is clearly centered away from zero, indicating that incidence scales nonlinearly with prevalence (Figure 2D). Overall, parameter estimates were largely consistent across genealogies, although there was considerable variation in the time of initial introduction of HIV into Detroit estimated from different trees. This is likely attributable to the large amount of variation in the root times inferred for different trees, as we inferred earlier times of introduction from trees with earlier root times.

**Table 2:** HIV parameter estimates.

Tree	*β_E_*	*β_C_*	*β_A_*	*α*×10^−4^	Initial Time
1	1.59 (1.43–1.77)	0.077 (0.006–0.187)	0.328 (0.146–0.653)	1.52 (0.51–4.35)	1975 (1973–1977)
2	1.69 (1.51–1.9)	0.079 (0.007–0.184)	0.318 (0.126–0.59)	1.72 (0.62–4.02)	1976 (1973–1979)
3	1.69 (1.39–1.99)	0.184 (0.02–0.539)	0.593 (0.151–1.26)	5.78 (1.43–11.8)	1976 (1973–1978)
4	1.51 (1.32–1.73)	0.079 (0.007–0.191)	0.316 (0.114–0.648)	1.55 (0.48–4.44)	1974 (1973–1976)
5	1.6 (1.29–1.92)	0.146 (0.016–0.395)	0.786 (0.235–1.64)	5.99 (1.91–11.1)	1973 (1973–1974)
6	1.69 (1.43–1.99)	0.114 (0.008–0.28)	0.543 (0.193–1.0)	3.75 (1.23–8.0)	1974 (1973–1975)
7	1.67 (1.45–1.91)	0.1 (0.012–0.224)	0.431 (0.155–0.823)	2.93 (1.21–5.57)	1974 (1973–1977)
8	1.87 (1.62–2.14)	0.095 (0.006–0.239)	0.521 (0.214–0.964)	4.06 (1.22–8.3)	1979 (1974–1981)
9	1.9 (1.51–2.31)	0.295 (0.025–0.636)	0.823 (0.247–1.74)	10.2 (2.76–17.5)	1980 (1974–1982)
10	1.52 (1.32–1.73)	0.08 (0.009–0.248)	0.433 (0.184–1.29)	2.5 (0.73–10.5)	1974 (1973–1976)

Median posterior and 95% credible intervals for parameters estimated from 10 HIV genealogies.

**Figure 3:**
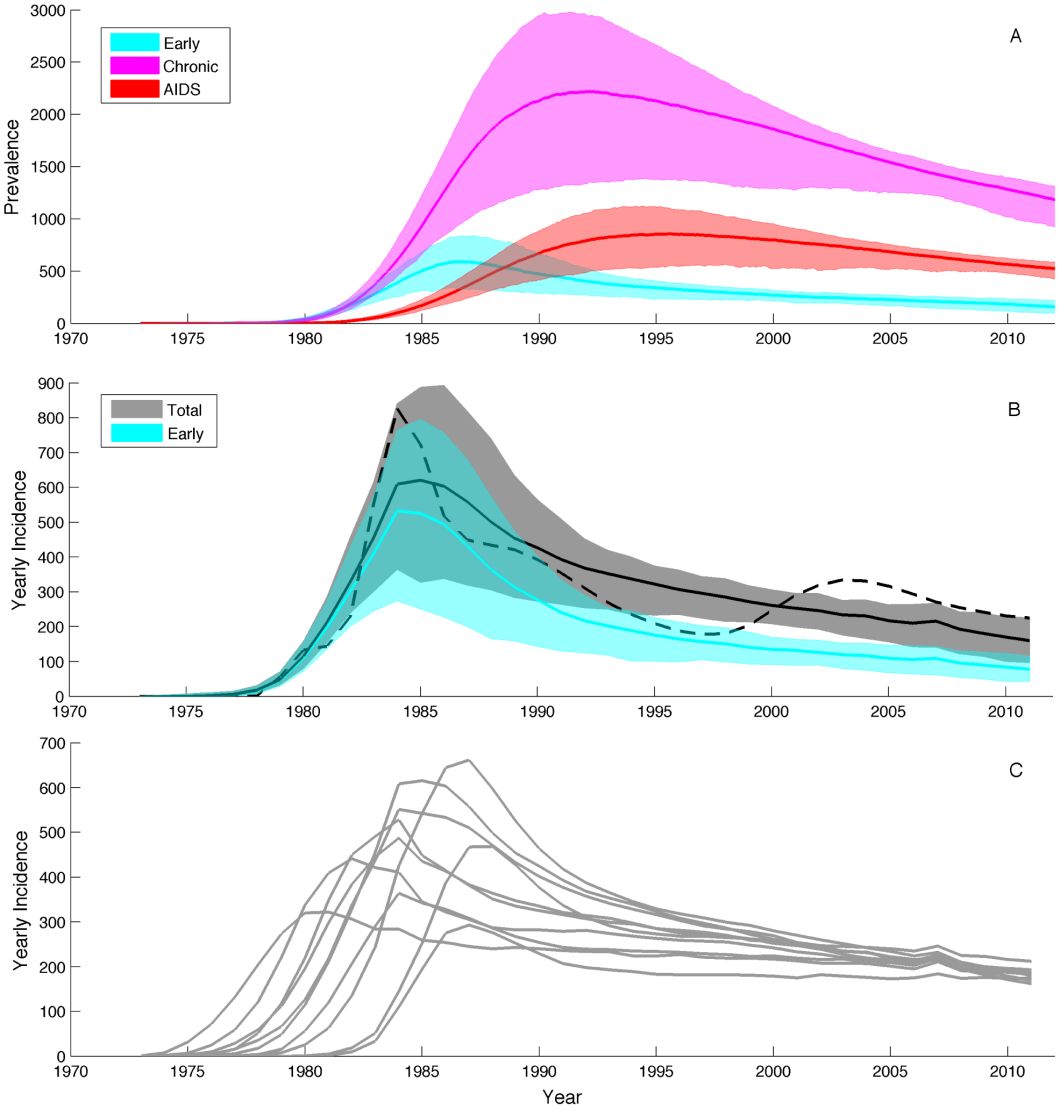
Population dynamics inferred from the Detroit HIV genealogies. (A) Stage-specific prevalence estimates from one genealogy with shaded regions showing the 95% credible intervals and lines the median of the posterior densities. (B) Estimated total yearly incidence and the estimated incidence attributable to early stage infections. The dashed black line shows the incidence back-calculated from Michigan Department of Community Health surveillance data. (C) Total incidence estimated from 10 randomly sampled HIV genealogies.

Stage-specific HIV prevalence inferred from the genealogies shows a predictable transition from most infections being in the early stage at the beginning of the epidemic to most infections being in the chronic or AIDS stages later in the epidemic (Figure 3A). This is expected given the longer duration of the chronic and AIDS stages. In general, our phylodynamic estimates of the epidemic dynamics closely track HIV incidence imputed from surveillance data from the beginning of the epidemic through the peak (Figure 3B). While our phylodynamic estimates do not capture the fluctuations in incidence that occur after 1990, there was nothing in our model that would allow us to reproduce this pattern, which likely results from complex changes in HIV treatment and behavioral changes [34]. Although there was also considerable variability in the population dynamics inferred from different genealogies, this variation occurs primarily during the early stages of the epidemic (Figure 3C). Again, this appears to be associated with uncertainty in the root times of trees; dynamics inferred from trees with earlier root times show an earlier rise and peak in incidence. After the epidemic peaks, the incidence estimated from different trees seems to converge on similar values.

Estimates of incidence attributable to each stage show that EHI contributed to most new infections at the beginning of epidemic when EHI prevalence was high (Figure 3B). After the epidemic peak, infections arising from EHI remains high proportional to EHI prevalence, consistent with the higher transmission rate we estimated for EHI. In the late 2000's, we estimated that between 40 to 50% of all new infections arise from EHI, indicating that early stage infections still play a major role in driving HIV transmission. These large estimates for number of new infections arising from EHI are consistent with the phylodynamic estimates of Volz *et al.* [34], who fit a more complex but deterministic epidemiological model to the same set of HIV sequences.

### Inferring population structure

**Figure 4:**
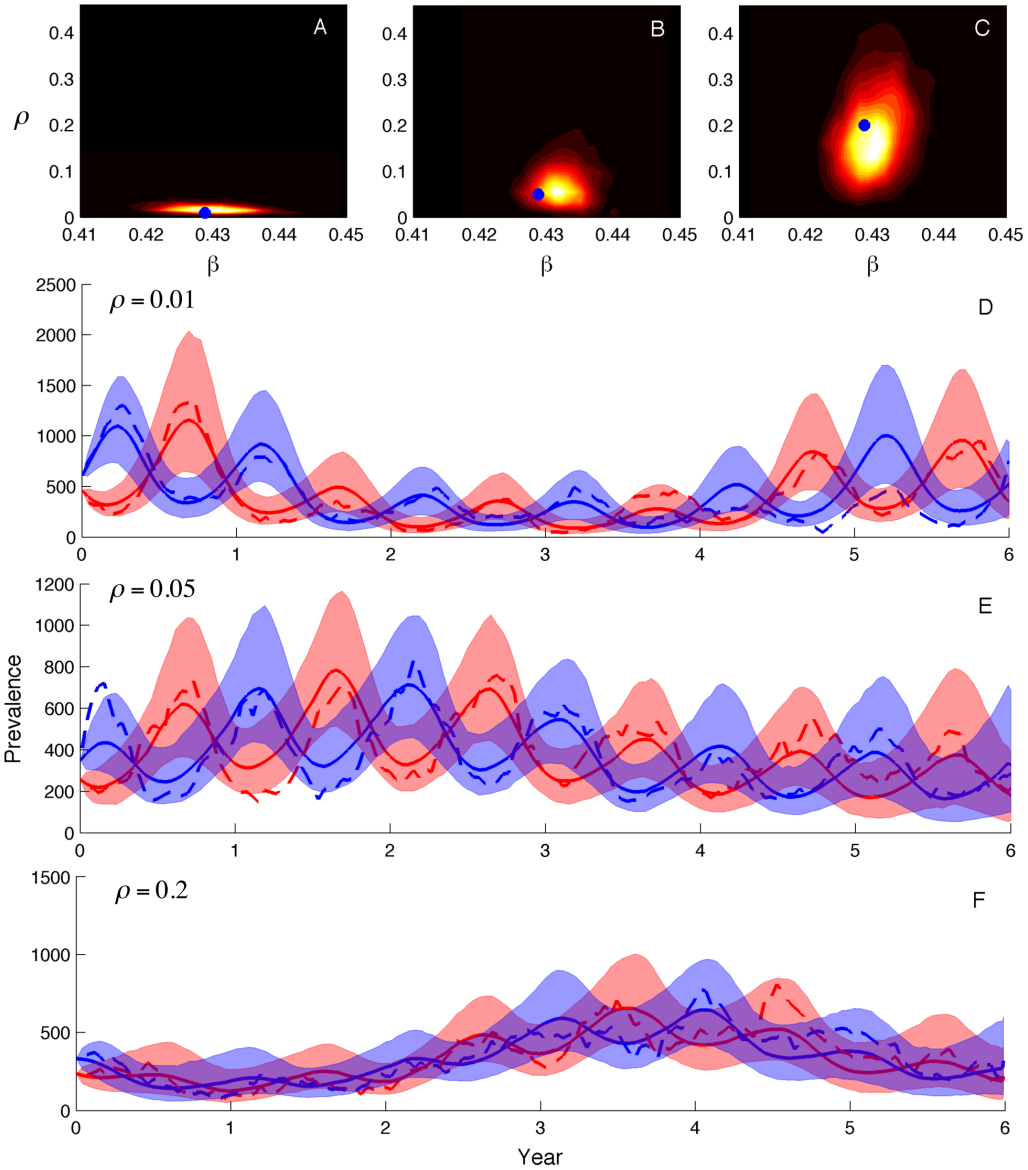
Parameter and prevalence estimates for the two-population model. Mixing rates between the two populations were varied from low (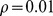), medium (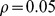) to high (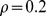). (A–C) Joint posterior densities for the transmission rate 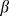 and the mixing parameter 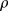. (D–F) Prevalence estimates for the two populations with the 95% credible intervals shaded and the posterior medians shown as solid lines. Dashed lines show the true prevalence. Initial conditions for the number of susceptible and infected individuals in each population were fixed at their true values for these simulations.

While our results for the three-stage model suggest that the PMMH algorithm works effectively and can be used to estimate key epidemiological parameters like HIV transmission rates, we were also interested in how much information genealogies contain about the structure of populations in general. To explore this question, we used the two-population model presented in (3), for which we can tune the strength of population structure by altering the mixing rate 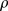 between populations. Mock genealogies were simulated under three values of 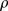: low (0.01), medium (0.05) and high (0.2). At 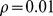, for example, about one in every one hundred transmission events occurs between populations. For all three values of 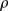, we were able to accurately infer the epidemiological parameters of interest and the population dynamics from the simulated genealogies (Figure 4 & Figure S3). While we can easily estimate 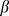 under all three demographic scenarios, the posterior densities become skewed towards increasingly high values of 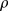 as mixing increases between the populations (Figure 4A–C). This indicates that it may be very difficult to obtain precise estimates of 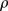 or other parameters pertaining to population structure when populations are only weakly structured.

**Figure 5:**
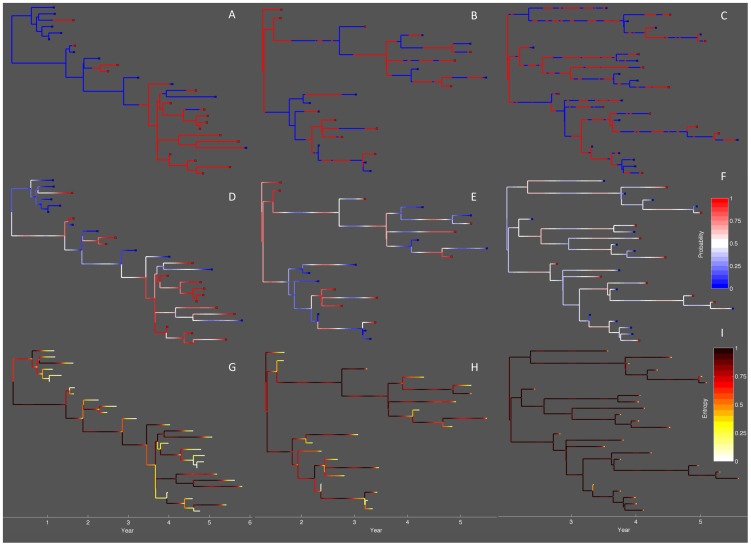
Genealogies simulated under different mixing rates. Mixing rates between the red and blue population were varied from low (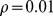), medium (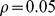) to high (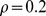). (A–C) The true lineage states mapped onto the genealogy. (D–F) Lineage state probabilities given with respect to the probability that the lineage is in the red state. (G–I) Entropy in the lineage states, which shows how much uncertainty there is in the lineage states. For each lineage 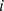, the entropy 
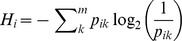
.

We can visually explore how much information a genealogy contains about population structure and pathogen movement by comparing the true lineage states to the computed lineage state probabilities. In Figure 5A–C, the true state of each lineage over time is mapped onto the genealogies. For ease of viewing, we only display a representative subtree of each genealogy. As expected, under low mixing lineages change states very slowly leading to a high degree of clustering among lineages sampled from the same population, whereas under high mixing lineages move rapidly between states and there is little clustering. We can then compare the true lineage states with the state probabilities computed under the median posterior values of the estimated parameters (Figure 5D–F). When 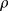 is low, the state of the lineages at the time of sampling is highly informative about the state of the lineage going into the past. However, when we increase 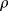 to 0.05, the state of the sampled lineages is less informative about the past states and we can see that the lineage state probabilities fluctuate seasonally according to the asynchronous dynamics between populations. When 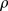 is high, the lineages move between states so rapidly that there is high uncertainty in the lineage states over the entire tree. This loss of information regarding the lineage states is readily observed by considering how the entropy, or uncertainty, in the lineage states changes going backwards in time (Figure 5G–H).

Visualizing the flow of information along the lineages in the trees shows how uncertainty in parameters like 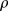 depends on how rapidly information about the lineage states decays. When 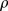 is low, lineages remain in the same state long enough that once a coalescent event is reached, information about the probable state of the lineages is still present. In this case, the probable states of the coalescing lineages provides additional information about the transmission event with respect to whether the transmission event occurred within or between populations. By combining information from coalescent events across the entire tree, we can then estimate the rates at which transmission occurs within and between populations. However, if all information about the past lineage states is lost before lineages coalesce, the observed coalescent events will no longer be informative about whether transmission occurred within or between populations and therefore parameters like 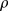 will be difficult to precisely estimate.

**Figure 6:**
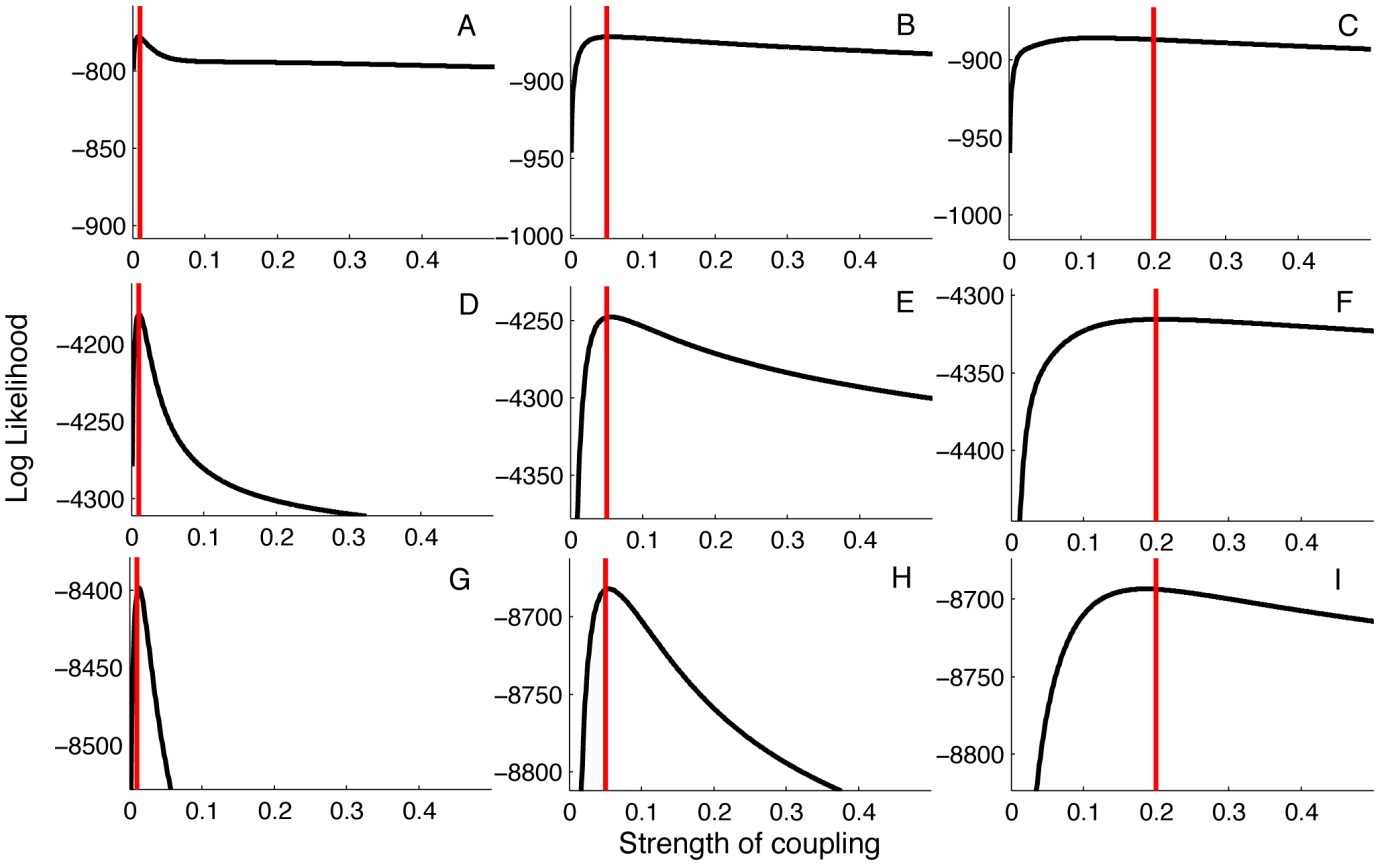
Likelihood profiles for the strength of coupling 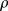 obtained from genealogies simulated under different values of 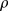. Red lines correspond to the true value of 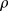. The likelihoods were computed from genealogies with 100 samples in (A–C), 500 samples in (D–F) and 1000 samples in (G–I). These sample sizes correspond, respectively, to approximately 0.2%, 1.0% and 2% of all infected individuals being sampled.

The preceding observations about uncertainty in lineage states suggest that it may be possible to estimate 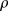 more precisely if we increase the number of sampled lineages. Increasing the sampling fraction will also increase the coalescent rate among lineages, thereby increasing the probability of lineages coalescing before all information about their probable state is lost. To test this idea, we simulated genealogies under the same three values of 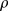 but varied the sample size. With a sample size of 100, the same as used above, we see that the likelihood is peaked around the true value of 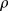 when mixing is low but the likelihood profile is fairly flat when mixing is high (Figure 6A–C). Increasing the sample size to 500 resulted in more curved likelihood profiles but the likelihood remains relatively flat with high mixing (Figure 6D–F). Doubling the sample size again to 1,000, the likelihood profiles show significant curvature for all values of 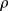 (Figure 6G–I). This suggests that while the sample size does play a significant role in determining whether parameters like 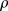 can be precisely estimated from genealogies, extremely large sample sizes may be required to estimate parameters pertaining to population structure when the population is only weakly structured.

## Discussion

The approach outlined in this paper allows for structured, stochastic epidemiological and other population dynamic models to be fit to genealogies in order to jointly infer past population dynamics and model parameters. We believe this to be an important step forward in the field of phylodynamics because many populations are structured in ways that could bias estimates of demographic parameters when using coalescent-based methods if population structure is not properly taken into account. Furthermore, unlike earlier methods for fitting structured coalescent models to genealogies (e.g. [7], [15]), our framework can accommodate non-equilibrium and nonlinear population dynamics and allows birth and migration rates to vary over time. We can also include stochasticity in our models when fitting them to data obtained from real populations, which may behave very differently than what would be expected under deterministic models. We can therefore fit the type of mechanistic population dynamic models typically used by epidemiologists and ecologists, which often include population structure, to genealogies.

As we have shown, fitting stochastic population dynamic models to genealogies through a structured coalescent model poses some challenges to statistical inference not normally dealt with in the statistical literature on fitting generic state space models to observational data. Under our structured coalescent models, the probability of a genealogy depends conditionally on both the population state variables as well as the states of individual lineages over time. However, going backwards in time, the probability that a lineage is in a certain state can strongly depend on the state that the lineage was sampled in at some future point in time. Particle filtering methods, which are widely used to fit state space models to other sources of data, can perform very poorly under these circumstances because the state of the system, in this case the lineage states, can depend strongly on the future states of the system. One strategy we initially tried was therefore to use a Gibbs sampling approach to iteratively sample from the conditional posterior densities of the population state and lineage state variables in independent steps to avoid the problem of having both forward and backward time dependencies in the model. Unfortunately, we found that such a Gibbs sampling strategy can be very inefficient and suffer from extremely poor MCMC mixing when there are strong correlations among the parameters and the lineage states. For example, in our two-population model, the mixing parameter 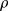 controls how rapidly lineages move between states and is thus highly correlated with the lineage states. If we update 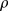 conditional on our current lineage states, the proposed value of 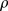 will need to be very close to the current value in order for the proposal to have high enough probability to be accepted conditional on the current lineage states. We therefore explore a potentially very large parameter space taking only small steps at a time.

Given these issues, we decided to use a modified version of the PMMH algorithm originally proposed by Andrieu *et al.* [22]. In this approach, we simply propose new parameter values each MCMC iteration and then run the particle filter to numerically integrate over the population state variables. To make the particle filtering algorithm as efficient as possible within each MCMC step, we allow for resampling by first weighting the particles according to the expected lineage state probabilities. Once we have run the particle filter forwards in time, we can then compute the true lineage state probabilities backwards in time and apply an additional round of importance sampling to correct for any bias introduced by using the expected lineage state probabilities. With the true lineage state probabilities of each particle, we can compute the coalescent likelihood of the genealogy while summing over all possible lineage states. We can therefore integrate over both the unobserved population state variables and the lineage state variables when computing the marginal likelihood of the parameter proposal. We thus have an efficient MCMC algorithm for sampling from the posterior density of the parameters without having to design independent proposals for the population states or the lineage states. The PMMH sampler therefore has a major practical advantage over other MCMC approaches that can be easily quantified. For the models considered in this paper, the PMMH algorithm typically converged in less than 100,000 iterations whereas for the Gibbs sampler we could run millions of MCMC iterations and still not converge. The efficiency of this approach will hopefully make it possible to also consider phylogenetic uncertainty in the future by sampling genealogies in addition to epidemiological parameters in the MCMC algorithm.

Whether or not the type of coalescent models considered here are appropriate for a particular pathogen is another important issue. The coalescent models assume that each infected host corresponds to a single pathogen lineage. If this were indeed always the case then coalescent events in the genealogy would always correspond to transmission events in the population. In reality, coalescent events will not occur instantaneously at transmission events but at some time before the actual transmission event because there will be a waiting time between when a lineage is transmitted and when it coalesces with another sampled lineage in the host. How closely the actual transmission event corresponds in time with the coalescent event will likely depend on the within-host dynamics of the pathogen [42]. For chronic viral infections like HIV where multiple lineages can persist within a given host for months or years, this may result in a large discrepancy in the timing of transmission and coalescent events. Nevertheless, a simulation study using a realistic distribution of within-host coalescent times for HIV found that the difference in timing between coalescent and transmission events was not sufficient to bias estimates of epidemiological parameters [34]. This may be due to the fact that a large fraction of HIV transmissions are due to recently infected individuals, in which case the within-host coalescent event cannot have occurred very long before the actual transmission event. A more principled approach to pursue in the future may be to impute the actual times of transmission conditional on the time of the coalescent events using information about within-host population dynamics. For example, additional information about pathogen population sizes over the course of a typical infection could provide an informative prior on waiting times between transmission events and coalescent events within hosts.

Another possible violation of the coalescent model occurs if sampled individuals have descendants that are themselves sampled, which can occur when samples are collected serially over time. The coalescent model implicitly assumes that when a new lineage is sampled, that lineage is sampled from a different host than any other lineage already in the genealogy. However, if a lineage is sampled from a host that has other sampled descendant lineages in the genealogy, then this results in a coalescent event in the tree that does not correspond to a transmission event in the population. A similar problem would arise if we unwittingly sampled more than one lineage from a single infected host. However this is likely to occur only if sampling is dense relative to prevalence over time. For example, if sampling is dense at the beginning and the end of an epidemic, then with a high probability hosts sampled at the beginning of the epidemic will likely have sampled descendants at the end of the epidemic. We acknowledge that the coalescent models used in this paper cannot adequately handle these types of situations, although for the HIV analysis it is unlikely that this is a serious problem seeing as all sequences were sampled in the recent past when prevalence was high. In cases where this is likely to be a serious problem, it may be worth developing metapopulation coalescent models, such as those introduced by Dearlove and Wilson [43], that allow hosts to be infected by more than a single lineage.

As our application to HIV showed, the PMMH algorithm allowed us to infer key epidemiological parameters like stage-specific transmission rates directly from genealogies. However, in the case of HIV, individuals stay in the same stage of infection for long periods of time relative to the timescale of the epidemic. The stage of infection of sampled individuals is therefore highly informative about the state of the lineage going into the past. Our experience with HIV may therefore not be representative of our general ability to infer parameters pertaining to pathogen transmission or movement in structured populations. In fact, our simple two-population SIR model revealed certain conditions under which it may be inherently difficult to estimate parameters relating to population structure. When lineages move between states rapidly due to transmission or migration any particular lineage is likely to have changed states multiple times before a coalescent event is reached, leading to high uncertainty about the state of lineage over the majority of the genealogy. This is somewhat analogous to the problem of site saturation in phylogenetic inference, where multiple transitions at a particular site along branches can render that site phylogenetically uninformative [44]. In the case of rapid transition rates among population states, observing the state of lineages at the time of sampling offers little or no information about the structure of the population because all information about the state of the lineage is quickly lost. Under these circumstances, it will be difficult to precisely estimate migration rates or other parameters relating to population structure from genealogies as we saw from the likelihood profiles of the mixing parameter in the two-population model, although it may be possible with many samples or a large sample fraction. This echoes earlier work on inference with structured coalescent models, where researchers have found it difficult to estimate migration rates from genealogies even without the complication of complex population dynamics [7], [45].

Although it may not always be possible to precisely estimate parameters relating to population structure from genealogies, we can imagine several cases in which the ability to fit mechanistic epidemiological models to genealogies that include population structure may be extremely useful. For example, our methods could be used to fit spatially structured models to genealogies of samples collected in different locations and could potentially complement recently developed phylogeographic methods that consider spatial structure but do not generally take into account local population dynamics at any particular location [46], [47]. For instance, incorporating both spatial and temporal dynamics could be important when the structure of a population is not static but changes over time due to changes in migration rates, which themselves may vary due to non-stationary population dynamics across locations. Our approach can also be applied in cases where sampling effort is distributed unevenly among populations so that the assumption of random sampling in unstructured coalescent models has obviously been violated. In this case, structured coalescent models can be used to control for non-random sampling as long as sampling is random within the subpopulations defined in the coalescent model. Finally, our methods can be applied to multi-host or vectored pathogens where lineages can move among different host or vector species. As shown in Rasmussen *et al.* [48] for the case of dengue, including the dynamics of both the host and vector populations in coalescent models may be necessary in order for population dynamics inferred from genealogies of vector-borne pathogens to be accurate.

We end by noting that the methods presented here can be used to fit epidemiological models to genealogies as well as other sources of data simultaneously. For example, we previously showed how unstructured epidemiological models can be fit to a genealogy and a time series of case reports simultaneously and it would be straightforward to extend the methods presented here to include time series or other observational data [21]. This could be especially helpful when certain parameters or aspects of the dynamics are difficult to infer from one data source but for which an alternative data source could be highly informative. For example, case report data may be aggregated over different subpopulations obscuring some of the heterogeneity present in the population but could be revealed by also considering information present in a genealogy. Consolidating data sources in this way will likely play an important role in epidemiological modeling in the future, especially as molecular sequence data become increasingly available and phylodynamic methods become integrated into modern epidemiology.

## Supporting Information

Figure S1 **Simulated epidemic dynamics for 100 stochastic realizations of the three-stage SIR model.** Total prevalence includes all three stages of infection.
(TIF)

Figure S2 **Representative time-scaled HIV genealogy from Detroit, Michigan.**
(TIF)

Figure S3
**Marginal posterior densities of the parameters in the two-population model with low mixing (** 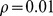 **) between populations.** Blue lines show the true values. The estimated parameters are: (A) the transmission rate 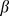; (B) the mixing rate 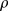; (C) the amplitude of seasonal forcing 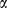; (D–E) the seasonal phases for the two populations 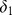 and 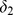. Similar estimates of the seasonality parameters were obtained with medium and high mixing between populations.(TIF)

Text S1 **Summary of the particle filtering algorithm for genealogies and details on how lineage state probabilities are computed.**
(PDF)
