# Supplementary figures and images for: Phylodynamic Inference for Structured Epidemiological Models
Source: PLoS Comput Biol. 2014 Apr 17;10(4):e1003570. doi: 10.1371/journal.pcbi.1003570 (PMC3990497; doi:10.1371/journal.pcbi.1003570)

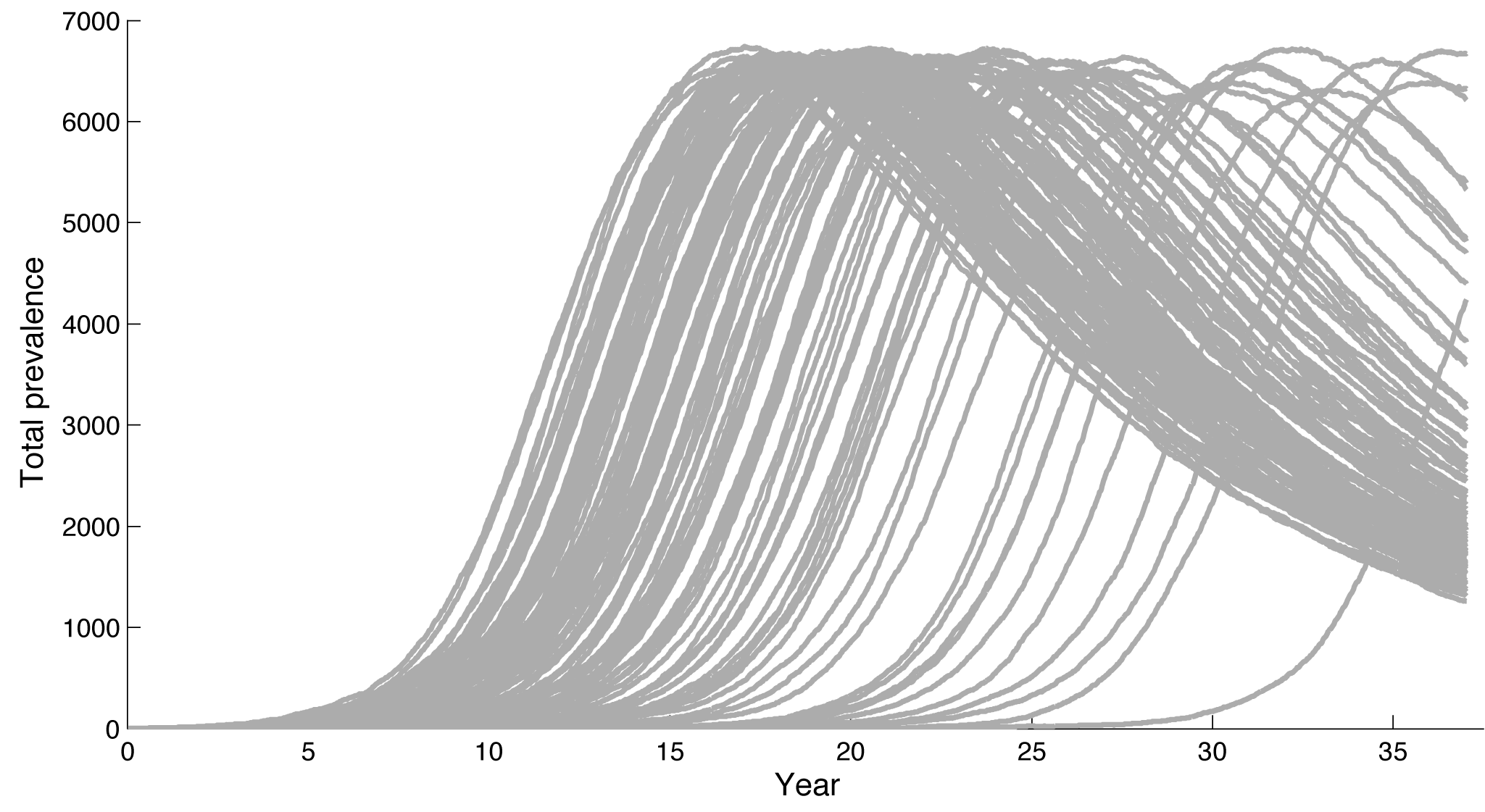

Supplement: Figure S1 — Simulated epidemic dynamics for 100 stochastic realizations of the three-stage SIR model. Total prevalence includes all three stages of infection. (TIF) [file pcbi.1003570.s001.tif]

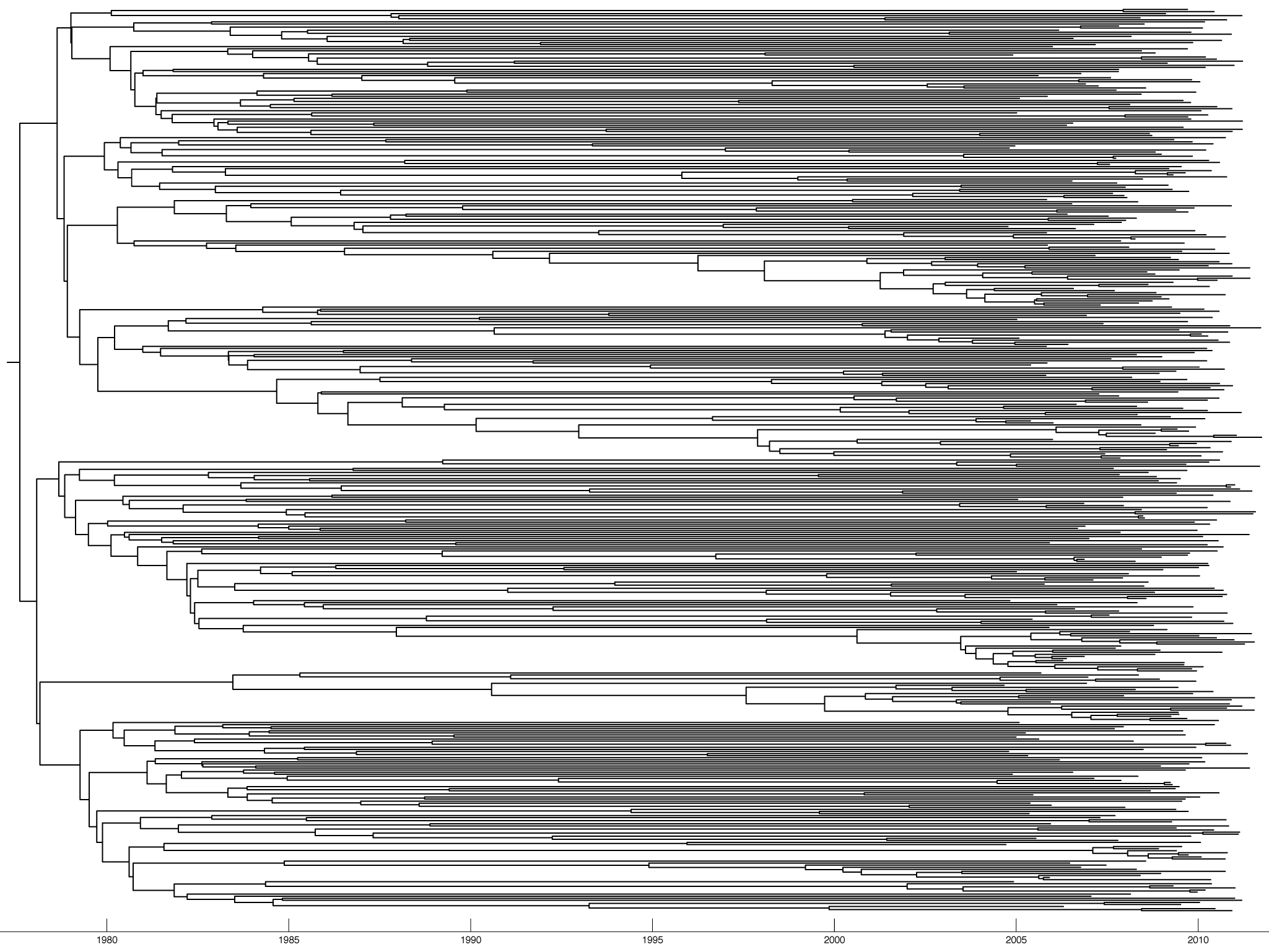

Supplement: Figure S2 — Representative time-scaled HIV genealogy from Detroit, Michigan. (TIF) [file pcbi.1003570.s002.tif]

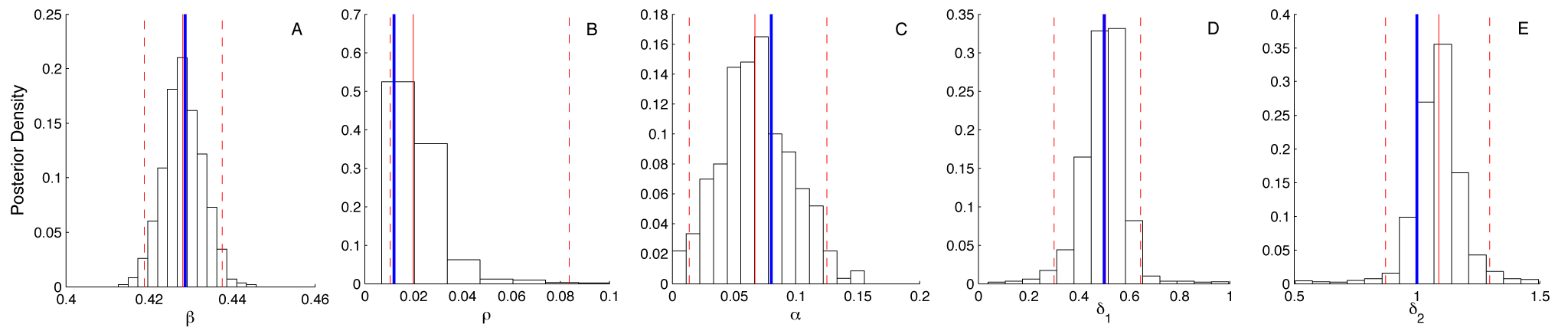

Supplement: Figure S3 — Marginal posterior densities of the parameters in the two-population model with low mixing ( ) between populations. Blue lines show the true values. The estimated parameters are: (A) the transmission rate ; (B) the mixing rate ; (C) the amplitude of seasonal forcing ; (D–E) the seasonal phases for the two populations and . Similar estimates of the seasonality parameters were obtained with medium and high mixing between populations. (TIF) [file pcbi.1003570.s003.tif]
