# Supplementary material for: Phylodynamic Inference for Structured Epidemiological Models
Source: PLoS Comput Biol. 2014 Apr 17;10(4):e1003570. doi: 10.1371/journal.pcbi.1003570 (PMC3990497; doi:10.1371/journal.pcbi.1003570)
Supplement: Text S1 — Summary of the particle filtering algorithm for genealogies and details on how lineage state probabilities are computed. (PDF) [file pcbi.1003570.s004.pdf]

Phylodynamic inference for structured epidemiological models

David A. Rasmussen[1,*], Erik M. Volz[2], Katia Koelle [1,3]
1 Biology Department, Duke University, Durham, NC, USA
2 Department of Infectious Disease Epidemiology, Imperial College London, London, UK
3 Fogarty International Center, National Institutes of Health, Bethesda, MD, USA
∗ E-mail: david.rasmussen@duke.edu

Text S1

Computing lineage state probabilities

As discussed in the main text, we require a way of computing the probability p_{ik} that a given lineage i is in a given state k at any point in time along the genealogy in order to compute the coalescent likelihood. The rates at which lineages transition between states through births and migrations are given in the F and G matrices, respectively. Given these transition rates, it is then possible to write down master equations for how the probability mass assigned to each state evolves backwards in time. As shown in Volz [1], the general form that these master equations take for any lineage i and state k is

$$\frac{d}{dt}p_{ik} = \sum_{l}^{m} \left(p_{il}\frac{g_{kl}}{y_l} - p_{ik}\frac{g_{lk}}{y_k} + p_{il}\frac{f_{kl}}{y_l}\frac{y_k - A_k}{y_k} - p_{ik}\frac{f_{lk}}{y_k}\frac{y_l - A_l}{y_l} \right), \tag{1}$$

where $A_k = \sum_{i \in \mathcal{A}} p_{ik}$; that is A_k is the expected number of lineages in state k in the genealogy at a given point in time. The first two terms in (1) give the probability mass gained or lost from the lineage transitioning in or out of state k through migration. The second two terms give the probability mass gained or lost from the lineage transitioning between states through a transmission event that was not observed as a coalescent event in the genealogy. In order for a lineage to transition from state l to state k in this way, there needs be a coalescent event between the lineage in state l and another lineage in state k that is not among the A_k sampled lineages in the genealogy so that it is not observed in the tree. The probability that the lineage in state k is not among the sampled lineages is $\frac{(y_k - A_k)}{y_k}$. This probability is then multiplied by the total rate at which lineages transition from state l to state k going backwards in time, $\frac{f_{kl}}{y_l}$, to get the total rate at which probability mass is gained by state k.

We also have to take into consideration how the lineage state probabilities get updated after a coalescent event. Given that lineages i and j coalesce, the parent lineage h may be either lineage i or j because we cannot observe from the tree which of the two lineages was the donor. To compute the probability that the parent lineage h was in state k when in transmitted, we therefore have to take into consideration all of the different ways h could have transmitted either lineage i or j. Conditioning on the current lineage state probabilities for lineages i and j, we therefore have

$$p_{hk} = \frac{1}{\lambda_{ij}} \sum_{l}^{m} \frac{f_{kl}}{y_k y_l} \left(p_{ik}p_{jl} + p_{il}p_{jk} \right). \tag{2}$$

Given these updates, we have everything needed to compute the lineage state probabilities over an entire genealogy.

Particle filtering with a genealogy

In Rasmussen *et al.* [2], it was shown how particle filters could also be applied to genealogies instead of standard observational data by using a coalescent model to relate the genealogy to the unobserved state variables. To briefly review the algorithm, the particle filter is run forward in time from time $t = 1$ to time $t = T$, sequentially updating the particle states x_t^j and assigning importance weights w_t^j for each particle

j at each time step. Particle states are updated at each time step by simulating from a proposal density $q(x_t^j|\bullet)$. Particle weights are then updated to reflect the posterior probability of each particle trajectory $x_{1:t}$ up to time t given the data observed up to time t, in this case the genealogy up to time t, $\mathcal{G}_{1:t}$. Therefore, at any time t, the weighted system of particles gives an importance sampling approximation to the density $p(x_{1:t}|\mathcal{G}_{1:t}, \theta)$. Once we reach time $t = T$, we sample a state trajectory $x^*_{1:T}$ by randomly selecting a particle according to the final normalized particle weights W_T to obtain a random sample from $\hat{p}(x_{1:T}|\mathcal{G}_{1:T}, \theta)$. We can also use the weights assigned to the particles to approximate the marginal likelihood of the parameters $p(\mathcal{G}_{1:T}|\theta)$.

Algorithm S1: The particle filter targeting $p(x_{1:T}|\mathcal{G}_{1:T}, \theta)$

1. Initialize the particle filter at time $t = 1$ with N particles.
   (a) Set x_1^j to initial values for all particles.
   (b) Assign normalized weights, $W_1^j = \frac{1}{N}$.
2. Run filter from $t = 2$ to $t = T$.
   (a) Propagate particles forward by drawing from the proposal density $q(x_t^j|\bullet)$.
   (b) Set $x_{1:t}^j = (x_{1:t-1}^j, x_t^j)$ for all particles.
   (c) Compute unnormalized weights,
   $$w_t^j = \frac{(w_{t-1}^j)p(\mathcal{G}_{t-1:t}|\theta, x_t^j)p(x_t^j|x_{t-1}^j, \theta)}{q(x_t^j|\bullet)}. \tag{3}$$
   (d) Normalize weights, so that $W_t^j = \frac{w_t^j}{\sum_{j=1}^N w_t^j}$.
   (e) If resampling at t, choose parent particle indexes a_t^j according to their weights, such that $p(a_t^j = k) = W_t^k$. Set $x_t^j = x_t^k$ and set $w_t^j = 1$. Otherwise, set $a_t^j = j$.
3. Sample $x^*_{1:T}$ from $\hat{p}(x_{1:T}|\mathcal{G}_{1:T}, \theta)$ by tracing the ancestry of one particle back through time.
   (a) Sample a single particle index k such that $p(k) = W_T^k$ and set $b_T^k = k$.
   (b) For $t = T - 1$ to $t = 1$, set $b_t^k = a_t^{b_{t+1}^k}$.
   (c) Set $x^*_{1:T} = x_{1:T}^{b_{1:T}^k}$.
4. Compute marginal likelihood estimate
   $$\hat{p}(\mathcal{G}_{1:T}|\theta) = \prod_{t=1}^{T} \frac{1}{N} \sum_{j=1}^{N} w_t^j. \tag{4}$$

Note that we have left the exact form of the proposal density $q(x_t^j|\bullet)$ unspecified in lack of an ideal proposal density. Nevertheless, we can update the particle states by simulating directly from the epidemiological process model $p(x_t|x_{t-1}, \theta)$ [3,4]. In this case, the weighting function simplifies to

$$w_t^j = (w_{t-1}^j)p(\mathcal{G}_{t-1:t}|\theta, x_t^j). \tag{5}$$

This has the fortuitous result that the term $p(x_t^j|x_{t-1}^j, \theta)$ does not appear in the weighting function so that we do not need to compute these transition densities explicitly, which is often not possible for continuous-time, nonlinear epidemiological models.

The particle filtering algorithm also allows for resampling to occur at the end of each time step, which is often necessary to ensure the practical feasibility of the algorithm. Resampling removes unpromising particle trajectories before we reach time T by replacing particles with low weights, and therefore very likely low posterior probabilities, with particles with high weights. However, it is often unnecessary and computationally wasteful to resample after each time step, especially if most particles have high unnormalized weights or there is little variance in weights across the particle population [5]. For this reason, we allow for adaptive resampling by making sampling after each step of the algorithm optional and generally resample as infrequently as possible. However, if we do resample, it requires us to track the ancestry of each particle in the population so that we can sample a single particle state trajectory at time T. We do this by recording the parent index a_t^j of each particle in the population at each time step. At time T, we choose a single particle index k and can trace that particle's ancestry back through time by setting $b_t^k = a_t^{b_{t+1}^k}$ for all times $t < T$. Thus $b_{1:T}^k$ gives the ancestral lineage of particle k in that b_t^k gives the index of the ancestor of particle k at time t. The state trajectory associated with particle k is then $x_{1:T}^{b_{1:T}^k}$.

References

1. Volz E (2012) Complex population dynamics and the coalescent under neutrality. Genetics 190: 187-201.

2. Rasmussen D, Ratmann O, Koelle K (2011) Inference for nonlinear epidemiological models using genealogies and time series. PLoS Comput Biol 7.

3. Ionides EL, Bretó C, King AA (2006) Inference for nonlinear dynamical systems. Proc Natl Acad Sci USA 103: 18438-43.

4. Cappe O, Godsill S, Moulines E (2007) An overview of existing methods and recent advances in sequential Monte Carlo. Proc Inst Electr Elect 95: 899-924.

5. Doucet A, Johansen A (2009) A tutorial on particle filtering and smoothing: Fifteen years later. Handbook of Nonlinear Filtering : 656-704.
